# Operational Status Assessment and Trend Prediction of Francis Turbine Generator Unit Shaft System Driven by Vibration and Swing Signals

**DOI:** 10.3390/s26165214

**Published:** 2026-08-17

**Authors:** Li Zhang, Shubo Qin, Zhiguo Feng, Jun Wang, Huqiang Sun, Simon X. Yang, Xiaobing Liu, Kun Yang

**Affiliations:** 1 Key Laboratory of Fluid and Power Machinery, Ministry of Education, Xihua University, Chengdu 610039, China; 2 CHN ENERGY Dadu River Repair & Installation Co., Ltd., Chengdu 610041, China; 3 Dadu River Pubugou Hydropower General Plant, China Energy Investment Group Co., Ltd., Ya’an 625304, China; 4 CHN ENERGY Dadu River Production Command Center, Chengdu 610041, China; 5 College of Engineering, University of Guelph, Guelph, ON N1G 2W1, Canada; 6 China Petroleum Engineering & Construction Corporation Southwest Company, Chengdu 610031, China

**Keywords:** vibration and swing signals, Francis turbine generator unit, shaft system, condition assessment, trend prediction, health monitoring

## Abstract

The operational reliability of shaft systems in hydropower units has become increasingly critical as these units are frequently engaged in grid regulation under new power systems. This paper presents a sensor-driven method for operational status assessment and trend prediction of Francis turbine generator unit shaft systems using vibration and swing signals. Time domain features are extracted from the sensor-acquired signals to construct a multi-dimensional quantitative index system for characterizing the operational state, and a combined Entropy Weight–Coefficient of Variation–TOPSIS model with dynamic health thresholds is established for adaptive condition assessment. To address the nonlinear and non-stationary characteristics inherent in such signals, a decomposition–prediction–reconstruction fusion framework is developed, incorporating Variational Mode Decomposition (VMD) for signal decomposition and noise reduction, iTransformer for capturing global multi-variable interactions, and Bidirectional Long Short-Term Memory (BiLSTM) for bidirectional temporal feature extraction. The hybrid model achieves a coefficient of determination R^2^ of 0.9845 on complex vibration and swing signals, demonstrating its superior prediction capability. Based on the prediction results, health scores and dynamic thresholds are calculated to perform trend analysis and health early warning. A case study is conducted using real-world monitoring data from a 306 MW Francis turbine unit. The results demonstrate that the proposed method effectively characterizes the shaft system operational state, achieving a closed-loop integration from condition monitoring to fault diagnosis and predictive maintenance. The operational status assessment and trend prediction analyses are in good agreement with actual operating conditions, providing reliable technical support for the intelligent health management of hydropower units.

## 1. Introduction

In the context of new power systems, hydroelectric turbine units serve as core equipment for grid stability regulation, and their operational reliability directly influences grid security and economic performance [1]. The shaft system, as the critical component responsible for energy transmission and mechanical load bearing, operates under complex and variable conditions and is vulnerable to the coupled effects of hydraulic, mechanical, and electromagnetic excitations, resulting in varying degrees of performance degradation. During actual operation, abnormal vibration swing caused by phenomena such as shaft misalignment has become one of the major contributing factors to unit shutdowns and even catastrophic failures [2]. Therefore, accurate status assessment and trend prediction of the shaft system are of considerable practical significance and engineering value for promoting the transition from corrective maintenance to predictive maintenance [3].

**Figure 1:**
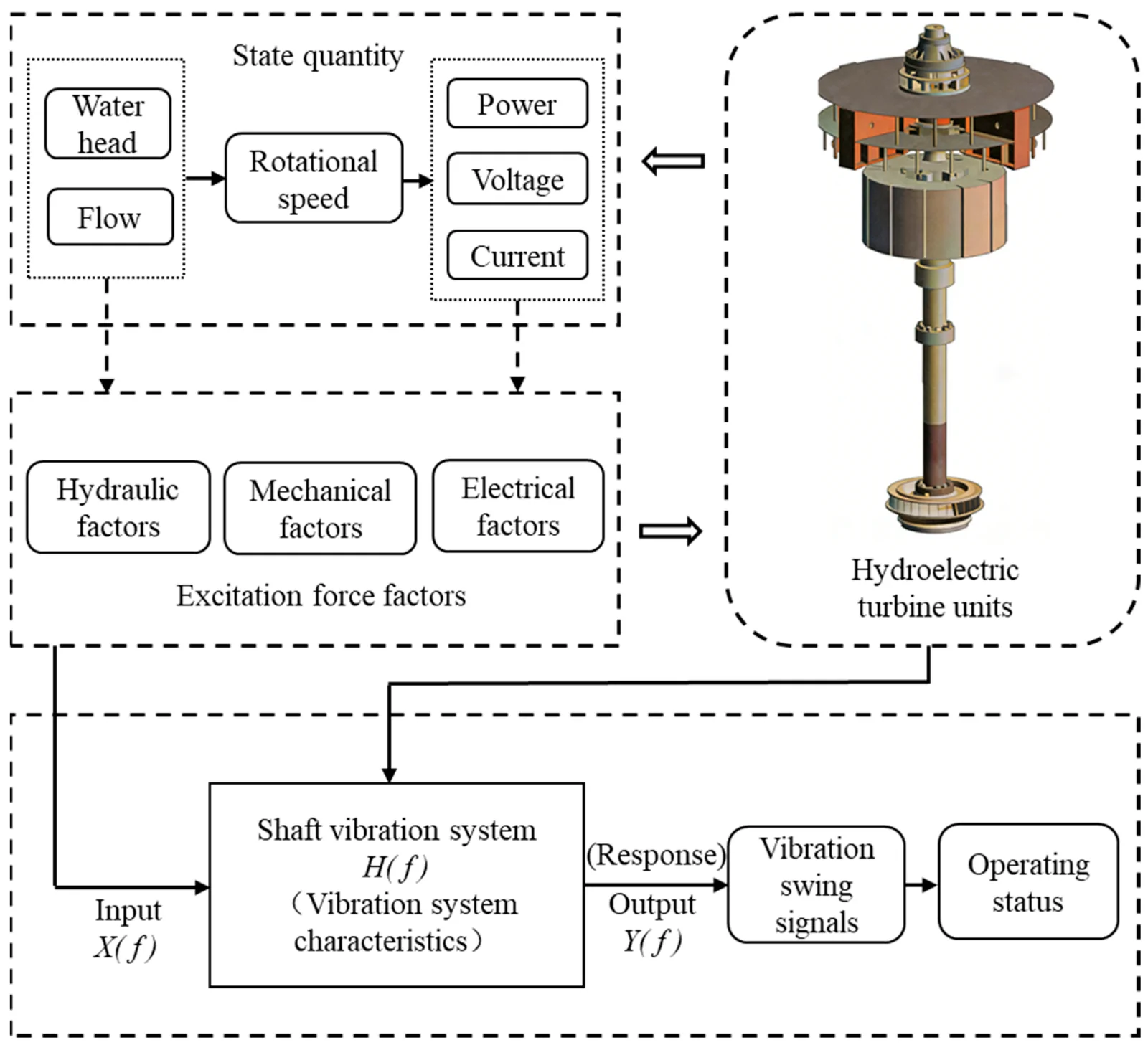
Schematic diagram of factors affecting vibration swing and the signal conversion process.

As shown in Figure 1, vibration and swing signals are key monitoring parameters that directly characterize the mechanical dynamic properties of the shaft system and contain abundant information regarding its operating status and health status [4,5]. Compared with slowly varying parameters such as temperature, these signals are more sensitive to early changes in shaft condition. Therefore, they are widely regarded as the most informative signal source for shaft system state assessment and trend prediction.

Regarding status assessment, traditional methods mainly build indicators from a single feature and depend strongly on fixed thresholds [6]. Ji et al. [7] used self-attention energy entropy to describe shaft system degradation; Liu [8] assessed component reliability by using the association between status features and reliability; Zhang et al. [9] developed a health model through sparse Gaussian process regression and used parameter deviation as an indicator; Wang et al. [10] extracted image differences from signals to develop indicators and determined vibration limits according to related standards [11].

For trend analysis based on vibration swing signal prediction, current studies show insufficient ability to model long time series [12,13] and limited extraction of multi-timescale features [14], which limits further improvement in prediction accuracy. In studies on vibration swing signal prediction, Chen [15] used a Gaussian Copula to select related parameters and built a DALSTM model; Hua [16] and Liu et al. [17] enhanced prediction accuracy by combining improved EMD with LSTM and by introducing a waveform extension method, respectively; Lu et al. [18] optimized VMD parameters and then combined the optimized decomposition method with LSTM. For condition and fault prediction, Fu et al. [19] proposed an EEMD–LSTM method for degradation degree prediction; Luo and Wu [20] integrated LSTM with DBN for fault prediction; Song et al. [21] included degradation indicators and used a VMD–Informer model to realize both assessment and prediction; He et al. [22] produced degradation degrees using MLP and then input them into an Attention–BiLSTM model for prediction. However, these studies usually lack further use of signal prediction results [23], such as trend analysis of operating status. In addition, the data amount and feature information available for model learning during prediction are still limited, and the generalization ability of the models still needs to be improved [24,25].

In summary, existing research methods still face three core challenges:

(1) Traditional approaches that rely solely on sampled data typically employ a limited set of indicators for health assessment. This not only makes it difficult to accurately determine the current condition of the shaft system but also hinders a comprehensive characterization of its complex operating states, potentially leading to the omission of degradation signatures or the generation of false alarms. Furthermore, the conventional practice of simply increasing the number of sensors overlooks the potential of multi-dimensional features embedded in the signals, while simultaneously incurring additional hardware costs.

(2) Over-reliance on fixed thresholds for sampled data or evaluation indicators can result in both missed detections and false alarms in health monitoring. Moreover, substantial disparities exist among different unit types, units of the same type, and even different monitoring systems. Methods that directly draw on prior experience struggle to accommodate the varying operating conditions of individual units, thereby reducing their engineering applicability.

(3) Much of the current research treats condition assessment and trend prediction as separate, isolated stages. That is, status assessment is conducted based on the current state and lacks predictive maintenance capability, while trend prediction is performed purely at the data level without further utilization of the predicted data. There remains a notable absence of systematic studies that feed prediction results back into the status assessment framework.

To address the above issues, this paper proposes a method for shaft system operating status assessment and trend prediction based on vibration and swing signals. The main contributions are as follows:

(1) Multidimensional time domain features are extracted from vibration and runout signals to construct an evaluation index system. A combined entropy weight–coefficient of variation–TOPSIS model is employed to calculate health scores, and dynamic health thresholds are introduced to enable adaptive status assessment.

(2) A decomposition–prediction–reconstruction integrated framework is developed, and a hybrid VMD–iTransformer–BiLSTM model is employed to realize accurate signal prediction.

(3) Based on the predicted signals, operating status trend analysis and health early warning are conducted, thereby forming a closed-loop technical framework from assessment to prediction. The effectiveness of the proposed approach is verified through a case study using real monitoring data from a hydropower station.

The rest of this paper is structured as follows. Section 2 describes the characteristics of vibration and swing signals and the development of the evaluation index system. Section 3 presents the operating status assessment method and the trend analysis strategy based on signal prediction. Section 4 provides a case study for validation of the proposed approach. Finally, Section 5 summarizes the main conclusions of the study.

## 2. Vibration and Swing Signals and Feature Representation

This section first examines the fundamental characteristics and engineering significance of vibration and swing signals. Subsequently, an evaluation index system based on time domain features is established to quantify the operating status of the shaft system, thus providing a data basis for subsequent status assessment and trend prediction.

### 2.1. Characteristics of Vibration and Swing Signals

**Figure 2:**
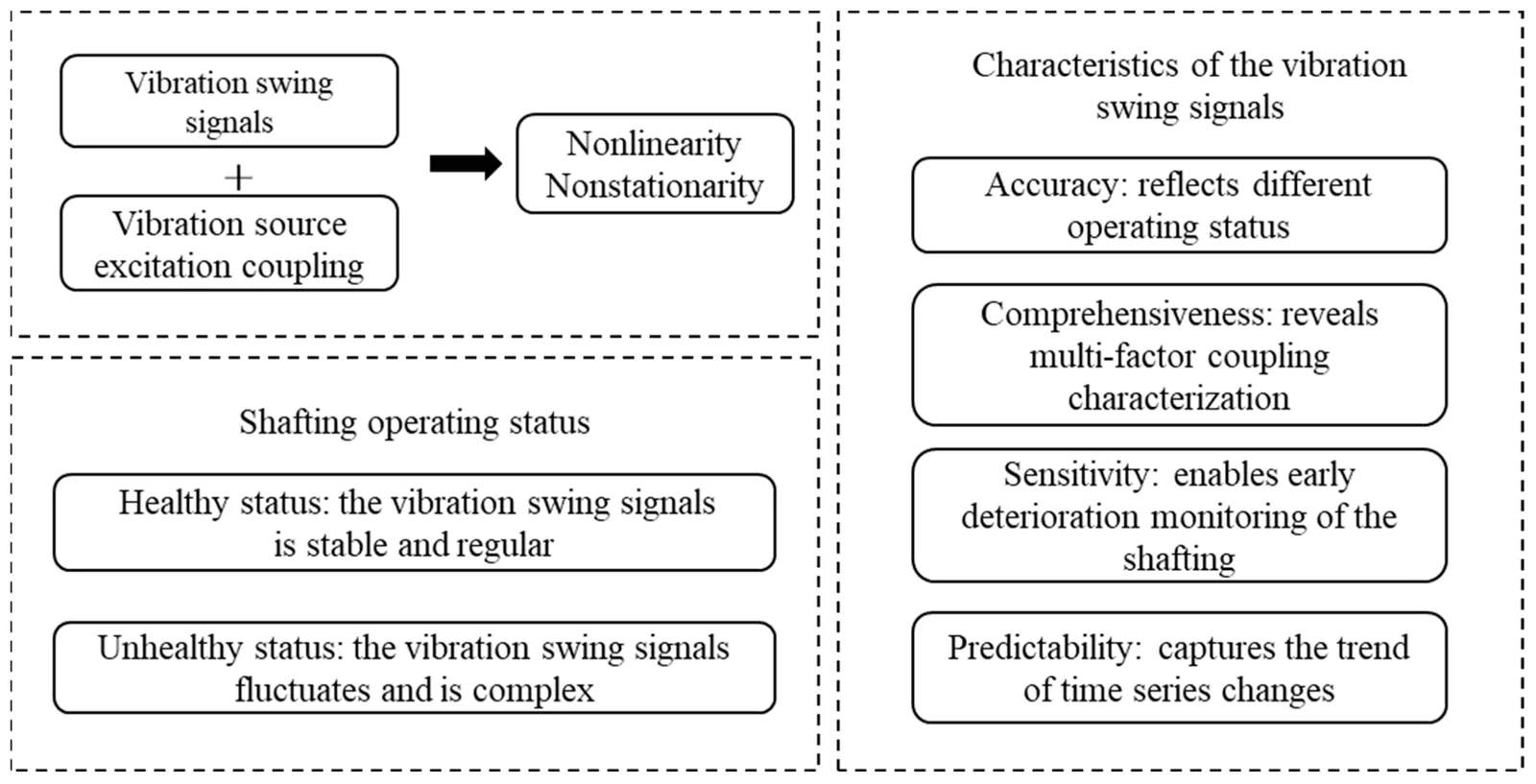
Characteristics of shaft system operational status and vibration and swing signals.

Vibration and swing signals exhibit characteristics of multi-component coupling and complex evolutionary behavior, and are closely associated with excitation sources. Besides their inherent nonlinear and non-stationary properties, these signals possess high accuracy in reflecting different operating states, comprehensiveness in representing multi-factor coupling effects, sensitivity to early shaft degradation, and predictability in temporal variation patterns [26,27]. Therefore, healthy, and abnormal shaft system conditions can be directly reflected through vibration and swing signals. The main signal characteristics are presented in Figure 2.

**Figure 3:**
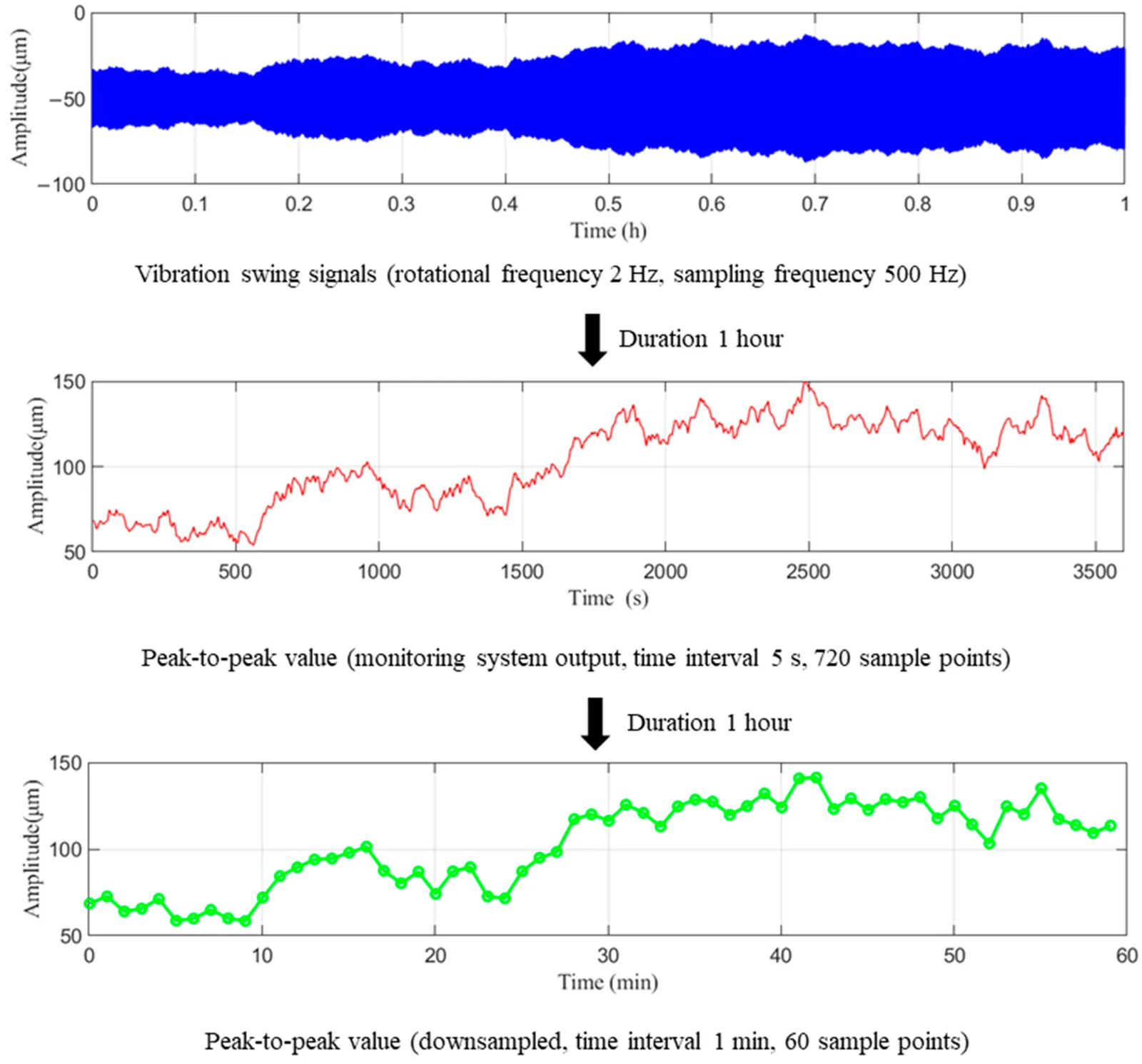
Schematic of peak-to-peak down-sampling of vibration and swing signals.

As vibration and swing signals are directly collected by sensors and their frequencies typically range from hundreds of Hz to kHz, a very large volume of data is generated, resulting in considerable storage requirements [3]. However, in practical engineering applications, peak-to-peak values of the signals, usually recorded at second-level intervals, are commonly used as the basic data form [28,29]. After data segmentation, assessment samples are generated for long-term health evaluation and early warning purposes. A schematic illustration of peak-to-peak down-sampling of vibration and swing signals is shown in Figure 3.

### 2.2. Construction of Operating Status Indicators Based on Feature Extraction from Vibration and Swing Signals

The operating status indicators are established according to the above analysis and relevant literature [30]. As the most direct descriptors of vibration and swing signals, time domain features can reflect shaft health conditions rapidly and intuitively. This gives them a clear advantage in the early warning of sudden faults. Time domain feature extraction from vibration and swing signals provides multidimensional information capable of characterizing shaft system status from different perspectives, which can then be used for health assessment. In this study, vibration and swing signals adopted for status assessment are divided into daily sub-samples, and time domain feature analysis is carried out for each sample. The extracted features are listed in Table 1.

To remove dimensional influences, the original indicator values are normalized using the min–max normalization method:

(1) $${a^{\prime }}_{ij}=\frac{a_{ij}-min\left(a_{j}\right)}{max\left(a_{j}\right)-min\left(a_{j}\right)}$$

where $a_{ij}$ represents the *j*th indicator value of the *i*th sample, and ${a^{\prime }}_{ij}$ represents the normalized value.

## 3. Operating Status Assessment and Trend Prediction

Status assessment and trend prediction of shaft systems are important technologies for ensuring the safe and stable operation of hydroelectric turbine units. Therefore, both aspects are addressed in this section. To address the limitation that a single weighting method cannot fully represent the information contained in the data, a combined entropy weight–coefficient of variation weighting strategy is proposed and integrated with the TOPSIS method for score calculation. To solve prediction difficulties caused by the nonlinear, non-stationary characteristics of vibration and swing signals and long time series data, a hybrid VMD–iTransformer–BiLSTM prediction model is developed. These two modules together establish an integrated assessment and prediction framework, providing technical support for shaft system condition monitoring and early warning.

### 3.1. Shaft System Status Assessment Based on the Combined Entropy Weight–Coefficient of Variation–TOPSIS Model

Operating status assessment is quantified using multidimensional indicators. To determine indicator weights, a combined weighting approach integrating the entropy weight method [31] and the coefficient of variation method [32] is introduced. This strategy simultaneously considers entropy-based dispersion and relative variability of indicators, thereby overcoming an important limitation of the traditional TOPSIS method [33], in which all indicators are assumed to have equal importance without considering their actual significance.

#### 3.1.1. Status Assessment Modeling

(1) Indicator Weights Based on the Entropy Weight Method

The entropy weight method is an objective weighting technique in which weights are assigned according to the characteristics of the data themselves. Within an evaluation system, entropy values are used to measure the dispersion degree of indicators. A smaller entropy value indicates greater dispersion and a larger amount of useful information carried by the indicator. Consequently, a higher weight is assigned to that indicator in the comprehensive evaluation process [31]. The entropy value *e_j_* and the weight $w_{j}^{EW}$ of the *j*th indicator are calculated as follows:

(2) $$\left\{\begin{array}{c} e_{j}=-\frac{1}{\ln n}\sum_{i=1}^{n}p_{ij}\ln p_{ij}\\ p_{ij}={a^{\prime }}_{ij}/\sum_{i=1}^{n}{a^{\prime }}_{ij} \end{array}\right.$$

(3) $$w_{j}^{EW}=\left(1-e_{j}\right)/\sum_{j=1}^{m}\left(1-e_{j}\right)$$

where $p_{ij}$ is the proportion of the *j*th indicator value for the *i*th sample.

(2) Indicator Weights Based on the Coefficient of Variation Method

The coefficient of variation method is an objective weighting approach in which weights are obtained by examining the internal variation of the data. This method assigns indicator weights according to the degree of variation; a larger variation in an indicator indicates higher importance and, therefore, a greater weight should be given [31]. The coefficient of variation *CV_j_* and the weight $w_{j}^{CV}$ of the *j*th indicator are calculated as follows:

(4) $$CV_{j}=\sigma_{j}/\mu_{j}$$

(5) $$w_{j}^{CV}=CV_{j}/\sum_{j=1}^{m}CV_{j}$$

where $\mu_{j}$ and $\sigma_{j}$ denote the mean value and standard deviation of the *j*th indicator, respectively.

(3) Synergy Test of the Combined Weights

The Kendall coefficient of concordance is used to examine the consistency between the two groups of weights [34]. The test statistic *W* is defined as:

(6) $$W=\frac{12\sum_{j=1}^{m}R_{j}^{2}-3k^{2}m{\left(m+1\right)}^{2}}{k^{2}m{\left(m-1\right)}^{2}}$$

where *k* represents the number of weight variables (*k* = 2), and *R_j_* denotes the sum of the ranks assigned to the *j*th indicator by the two methods. If the test is satisfied, the combined weight obtained from Equation (7) is used; otherwise, the weights are recalculated through the CRITIC method [35].

(4) Combined Weights

When the Kendall coefficient of concordance test is passed, only a small internal difference exists among the weight vectors. The combined weight can then be calculated using the weighted average method, which considers both information content and dispersion degree:

(7) $$w_{j}^{CO}=\left(w_{j}^{EW}+w_{j}^{CV}\right)/2$$

(5) TOPSIS Composite Score

The TOPSIS method scores samples by calculating their distances from the positive ideal solution and the negative ideal solution [33]. It uses relative closeness to represent the distance of each evaluation indicator from the reference values. After the maximum and minimum reference values are determined, the distance of each indicator from these references is calculated: values closer to the maximum receive higher scores, whereas those closer to the minimum receive lower scores. Let the normalized decision matrix be denoted as $Z^{\prime }$, then the weighted matrix *Z* is given by:

(8) $$Z_{ij}=w_{j}^{CO}{Z^{\prime }}_{ij}$$

The positive ideal solution $Z_{j}^{+}$ and the negative ideal solution $Z_{j}^{-}$ are defined as the maximum and minimum values of each indicator. The distances of the *i*th sample from the positive and negative ideal solutions, together with its health score *S_i_*, are calculated as:

(9) $$D_{i}^{+}=\sqrt{\sum_{j=1}^{m}{\left(Z_{ij}-Z_{j}^{+}\right)}^{2}},\;D_{i}^{-}=\sqrt{\sum_{j=1}^{m}{\left(Z_{ij}-Z_{j}^{-}\right)}^{2}}$$

(10) $$S_{i}=\frac{D_{i}^{-}}{D_{i}^{+}+D_{i}^{-}}\times 100$$

where *S_i_* ranges from 0 to 100, with higher values indicating more severe degradation.

(6) Dynamic Health Threshold

The shaft system health scores follow a normal distribution under the healthy status. A dynamic health threshold S’ is established based on the 3σ principle [36]. The threshold is computed using data prior to the current time point, and it is updated dynamically at each time step:

(11) $$\mu =\frac{1}{n}\sum_{i=1}^{n}S_{i},\;\sigma^{2}=\frac{1}{n}\sum_{i=1}^{n}{\left(S_{i}-\mu \right)}^{2}$$

(12) $$S^{\prime }=\mu +k\sigma$$

where μ and σ denote the mean and standard deviation of the health scores *S* computed from all samples up to and including the *i*th sample.

*S* remains within the dynamic threshold *S*′ under the normal operating status. When *S* consistently exceeds *S*′, the shaft system can be considered to have entered an unhealthy status, requiring close monitoring of its condition, as subsequent shaft faults may occur.

The main contribution of this section lies in integrating complementary foundational methods with an adaptive implementation that accounts for unit-specific differences, thereby addressing the shortcomings of existing engineering approaches.

#### 3.1.2. Status Assessment Procedure

**Figure 4:**
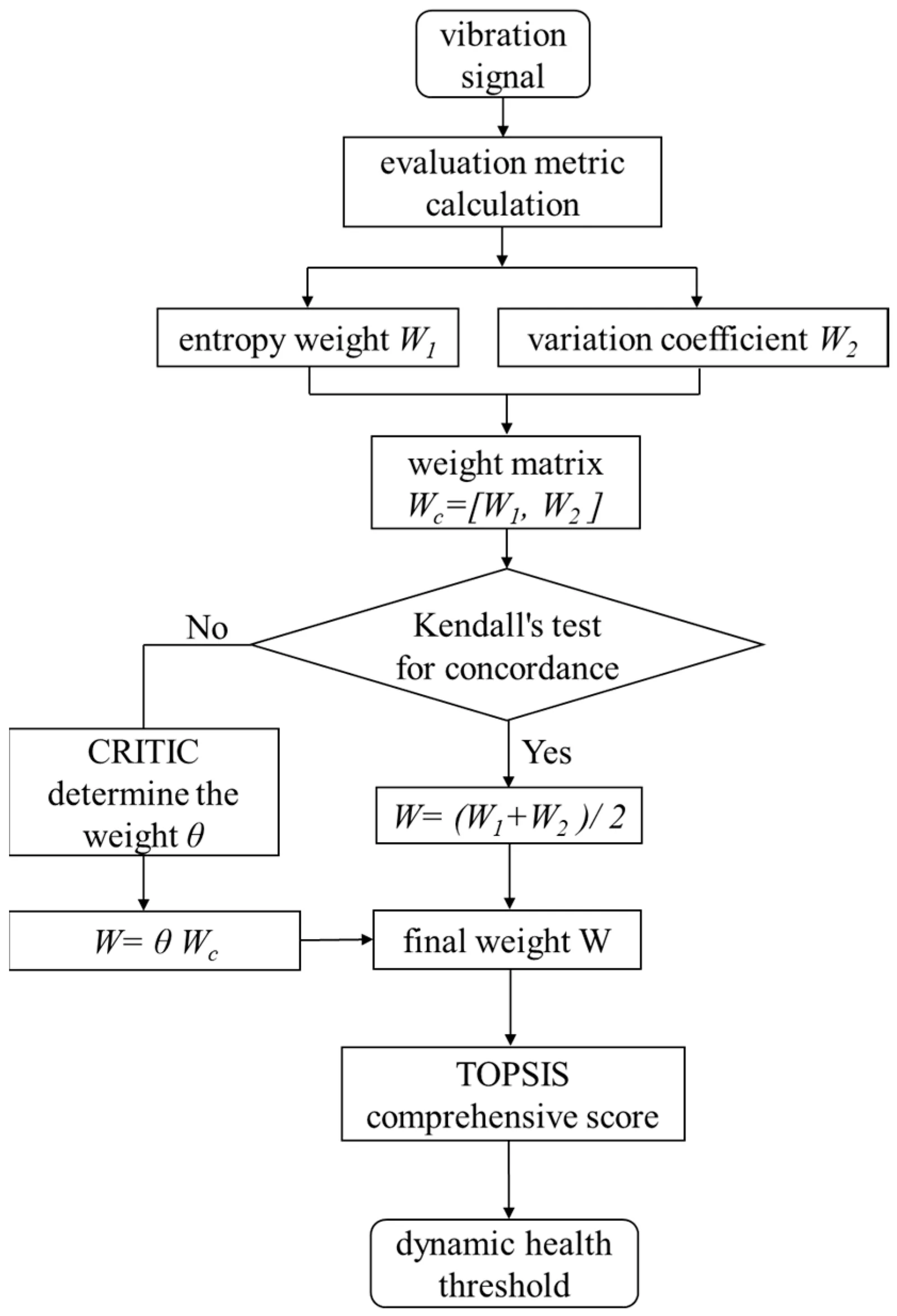
Flowchart of the entropy weight–coefficient of variation–TOPSIS assessment procedure.

In the status assessment procedure, vibration and swing signals are partitioned into daily samples, after which each indicator is calculated. The entropy weight method and the coefficient of variation method are used to derive two separate sets of indicator weights, which are then combined. During the combination process, the discrepancy between the two sets of weights is examined. Once the weights for each indicator are determined, the TOPSIS method is employed to compute the health scores. The corresponding steps are illustrated in Figure 4.

Step 1: Partition the vibration and swing signals into daily samples, extract 11 time domain feature indicators, and complete data normalization.

Step 2: Calculate the indicator weights using the entropy weight method and the coefficient of variation method, and determine the combined weights after passing the Kendall coefficient of concordance test.

Step 3: Compute the health score of each sample using the TOPSIS method.

Step 4: Establish a dynamic health threshold based on the 3σ principle to assess the operating status of the shaft system.

### 3.2. Prediction Modeling of Vibration and Swing Signals Based on VMD–iTransformer–BiLSTM

Vibration and swing signals serve as essential dynamic indicators that characterize the shaft system operating condition of hydroelectric turbine units. Their trend prediction is critically important for early fault warning and maintenance decision making.

#### 3.2.1. Hybrid VMD–iTransformer–BiLSTM Prediction Model

Vibration and swing signals exhibit pronounced nonstationarity, making it difficult for a single time series prediction model to capture their intrinsic variation patterns [37]. In this paper, these signals are conceptualized as a manifestation of complex dynamic behavior, in which the variation in any given component is jointly governed by the remaining components and associated features such as water head and active power [25,38]. Therefore, a hybrid VMD–iTransformer–BiLSTM prediction model is proposed, and its core idea is outlined as follows:

- VMD decomposes the non-stationary vibration and runout signals into a set of relatively stationary intrinsic mode functions (IMFs). It reduces the difficulty for the model in directly learning aliased features while mitigating the effect of noise.
- iTransformer employs an inverted attention mechanism to strengthen the global interactions among variables such as water head H and active power P and captures the complex coupling relationships.
- BiLSTM leverages its bidirectional architecture to incorporate both past and future information and captures bidirectional temporal dependencies within the sequence.

The combination of these three components fully utilizes their respective strengths in signal decomposition, multivariate interaction modeling, and bidirectional temporal feature extraction.

(1) Signal Decomposition Based on VMD

Variational mode decomposition (VMD) is a fully non-recursive signal decomposition method [39] that removes high-frequency noise through decomposition and reconstruction, yielding a denoised signal. For an input signal *x(t)*, VMD is formulated as a constrained variational problem in the time domain as follows:

(13) $$\left\{\begin{array}{c} \underset{m_{k},\omega_{k}}{\min}\left\{\sum_{k=1}^{K}{\left\|\partial_{t},\left[\left(\delta (t)+j/\pi t)*m_{i}(t\right)\right],e^{-j\omega_{k}t}\right\|}_{2}^{2}\right\}\\ s.t.\sum_{k=1}^{K}m_{k}(t)=x(t),\;k=1,2,\ldots ,K \end{array}\right.$$

where *K* denotes the total number of decomposition modes, $m_{k}$ is the *k*th mode component, and $\omega_{k}$ represents the *k*th center frequency after decomposition.

To solve this variational problem, a quadratic penalty term and a Lagrangian multiplier λ are introduced, and the alternating direction method of multipliers (ADMM) is employed to iteratively update the mode components $m_{k}$, $\omega_{k}$ and λ.

(2) iTransformer Multivariate Interaction

iTransformer [40] is a variant of Transformer [41] that uses only the encoder to simplify the structure and effectively extract global feature representations. Different from the traditional Transformer, which applies the attention mechanism along the time dimension, iTransformer transposes the embedding layer, regards each feature series as an independent token, and applies the attention mechanism and feed-forward networks along the variable dimension to learn nonlinear representations. Therefore, complex coupling relationships among multiple variables can be captured more effectively.

(3) BiLSTM-Based Bidirectional Temporal Feature Extraction

The Bidirectional Long Short-Term Memory network (BiLSTM) [42] processes sequential data in parallel through two independent LSTM layers, including a forward layer and a backward layer. Bidirectional temporal dependencies within the data can therefore be captured. Information flow in LSTM is controlled through forget, input, and output gates, which effectively alleviates the vanishing gradient problem commonly observed in conventional recurrent neural networks (RNNs) and supports the retention of useful information over long time periods [43].

#### 3.2.2. Trend Analysis Method for Shaft System Operational Status Based on Predicted Signals

**Figure 5:**
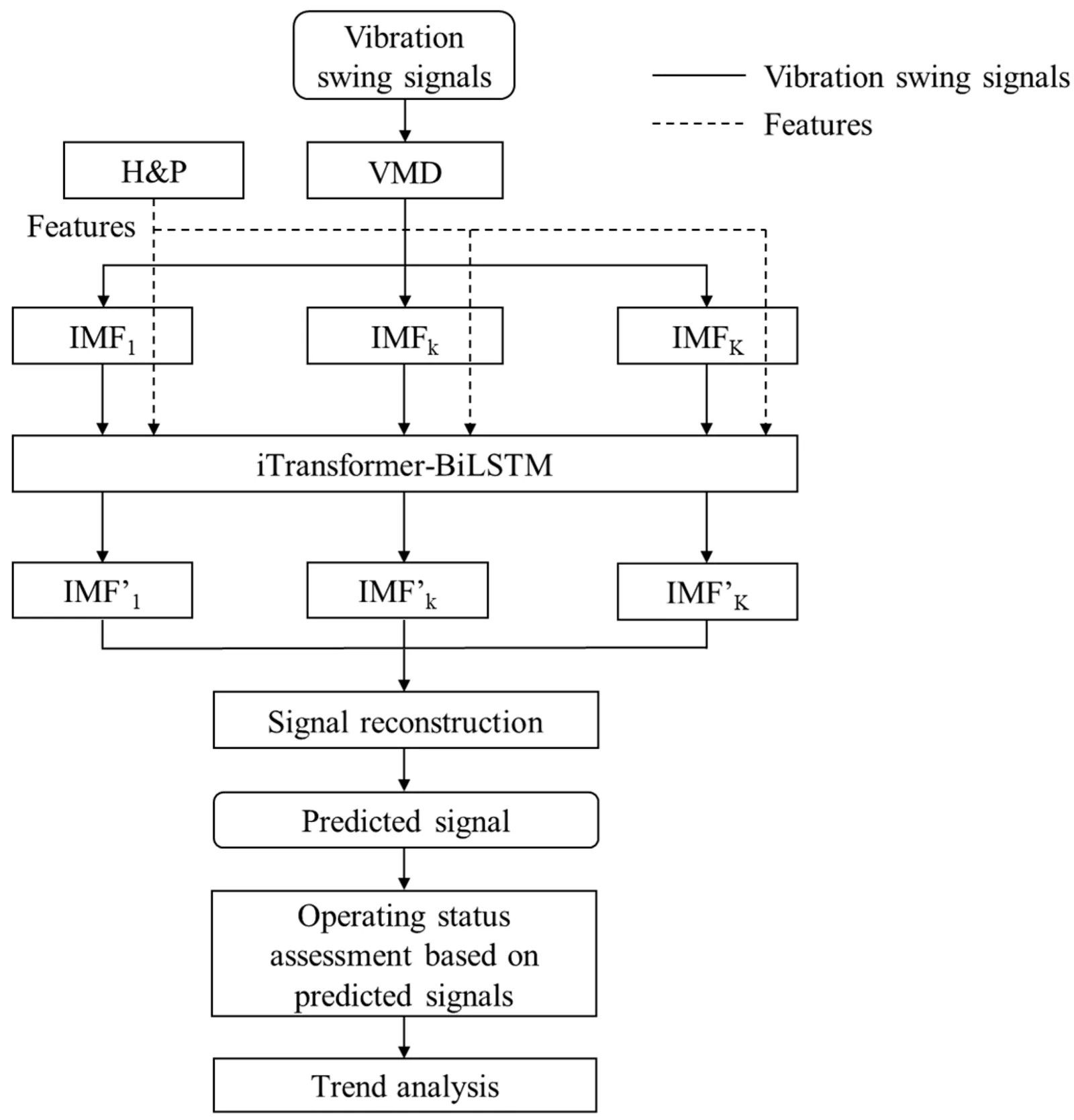
Trend analysis process of shaft system operational status based on predicted vibration and swing signals.

In this paper, a prediction framework for vibration and swing signals is constructed based on signal decomposition algorithms and deep learning models. The predicted results are further used to conduct trend analysis of the shaft system operating status. The overall process is shown in Figure 5.

Step 1: The original vibration and swing signals are decomposed by VMD to obtain a group of relatively stationary IMF components.

Step 2: Each IMF component, together with contextual variables such as water head and active power, is input into iTransformer to capture global interactive features among variables.

Step 3: The output sequences of iTransformer are transferred to the BiLSTM model to extract bidirectional temporal dependencies and generate the predicted IMF components.

Step 4: The predicted IMF components are reconstructed to obtain the complete forecasted vibration and swing signals. Then, the health scores and dynamic health thresholds are calculated from the signals to perform operating condition trend analysis.

This workflow divides the complex prediction task into several simpler subtasks, clearly reducing the difficulty of directly learning mixed features. It also weakens the effect of noise and improves the stability and robustness of the prediction model.

#### 3.2.3. Prediction Performance Evaluation Metrics

Five quantitative metrics are employed in this paper to evaluate prediction performance: mean absolute error (MAE), mean absolute percentage error (MAPE), mean squared error (MSE), root mean squared error (RMSE), and coefficient of determination (R^2^).

(14) $$MAE=\frac{1}{N}\sum_{i=1}^{n}\left|Y_{i}-{\hat{Y}}_{i}\right|$$

(15) $$MAPE=\frac{1}{N}\sum_{i=1}^{n}\left|\frac{Y_{i}-{\hat{Y}}_{i}}{Y_{i}}\right|\times 100\%$$

(16) $$MSE=\frac{1}{N}\sum_{i=1}^{n}{\left(Y_{i}-{\hat{Y}}_{i}\right)}^{2}$$

(17) $$RMSE=\sqrt{\frac{1}{N}\sum_{i=1}^{n}{\left(Y_{i}-{\hat{Y}}_{i}\right)}^{2}}$$

(18) $$R^{2}=1-\frac{\sum {\left(Y_{i}-{\hat{Y}}_{i}\right)}^{2}}{\sum \left(Y_{i}-\bar{Y}\right)}$$

where *N* is the number of set samples, $Y_{i}$ represents the actual value, $\bar{Y}$ denotes the mean of the actual values, and ${\hat{Y}}_{i}$ represents the predicted value.

## 4. Case Study

### 4.1. Dataset and Processing of Vibration and Swing Signals from a Hydropower Station

**Figure 6:**
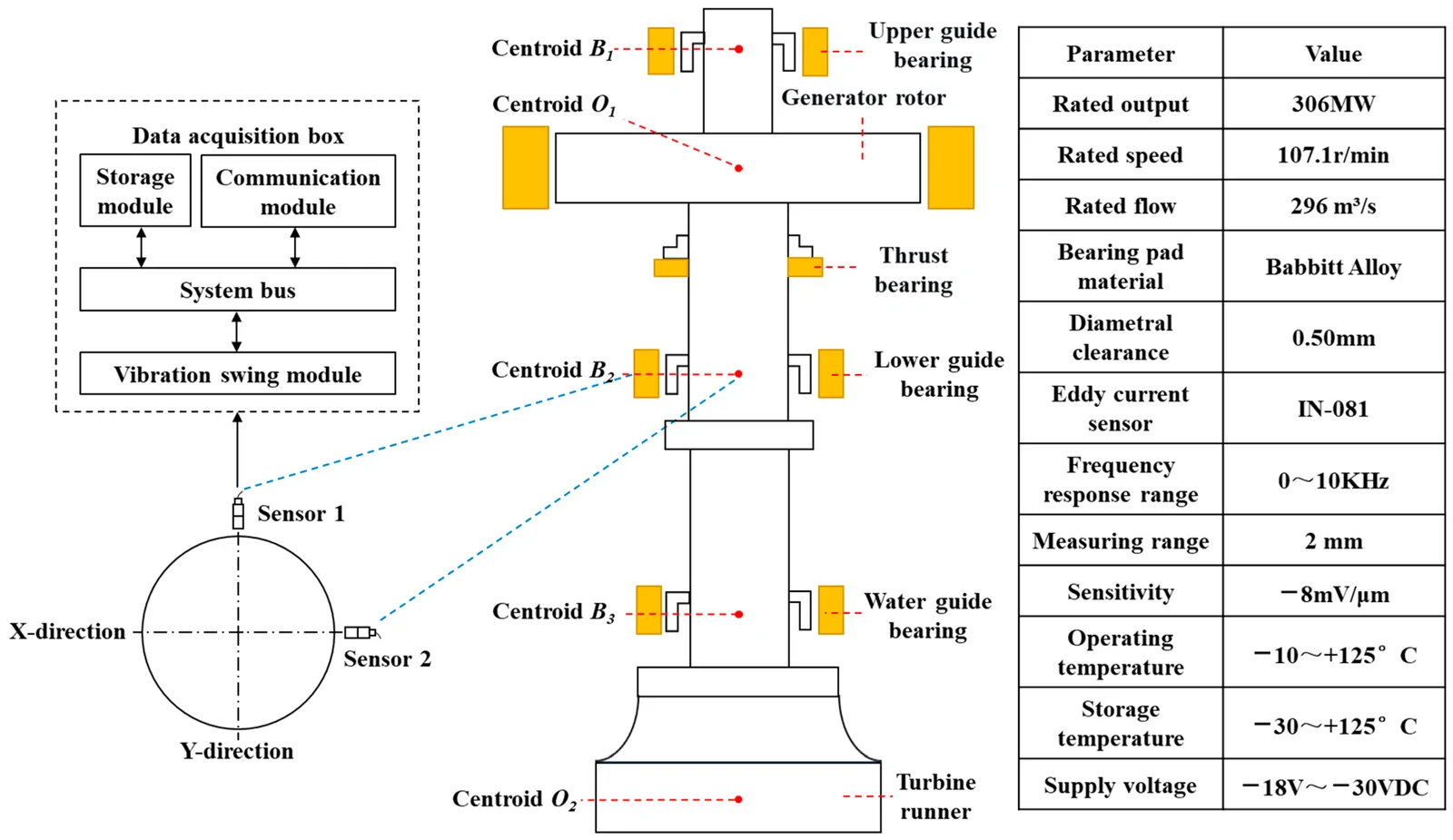
Equipment and data acquisition schematic diagram and parameters.

Monitoring data collected from a hydropower station between 15 September and 31 December 2023, are used in this study. The Francis turbine unit has a rated power of 306 MW and a rated speed of 107.1 r/min. The schematic diagram of the equipment and data acquisition, along with the relevant parameters, is shown in Figure 6.

The vibration and runout data are composed of the peak-to-peak values in the X direction of the lower guide bearing, and the sampling interval of the exported monitoring system data is about 5 s.

**Figure 7:**
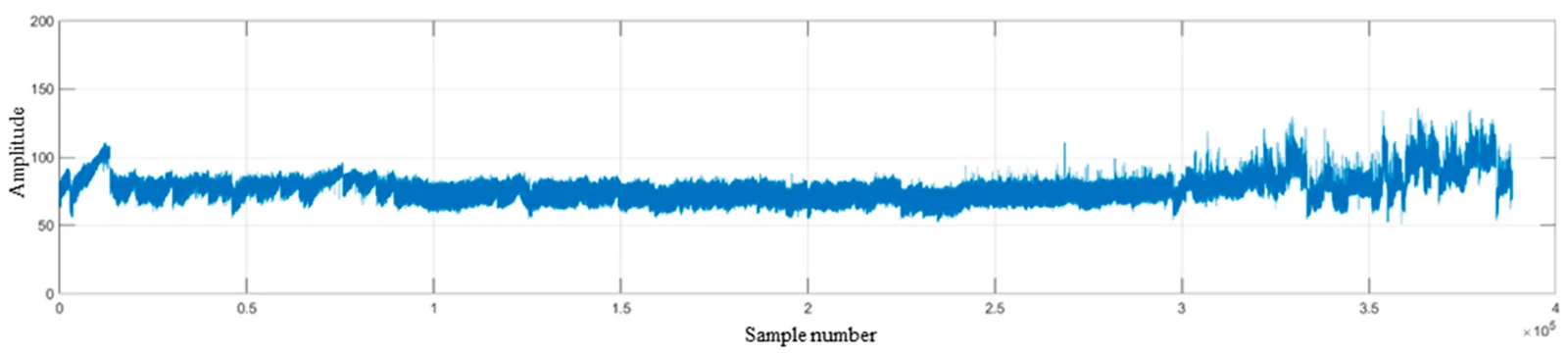
Peak-to-peak values in the X direction of the lower guide bearing for active power between 214.2 MW and 306 MW.

According to Table B.4 of GB/T 32584-2016 [11], the normal operating range of vertical Francis turbine units is defined as 70–100% of the rated output. Therefore, the peak-to-peak vibration and runout signals under normal operating conditions with active power between 214.2 MW and 306 MW were extracted. The filtered signal data curve is presented in Figure 7.

Data from start-up, shutdown, and low-power operation periods were removed. As shown in Figure 7, under normal operating conditions, the vibration and swing signals start to show obvious nonstationarity and fluctuations near the 300,000th sampling point. Further examination of the data indicates that this period corresponds to November 23, leading to a preliminary qualitative judgment that the health status of the shaft system may deteriorate clearly in late November. Meanwhile, the signal curve is discontinuous over the long term. To solve this problem and support accurate analysis, the dataset was divided into daily subsamples. It is found that the data within each day are basically a continuous time series.

**Figure 8:**
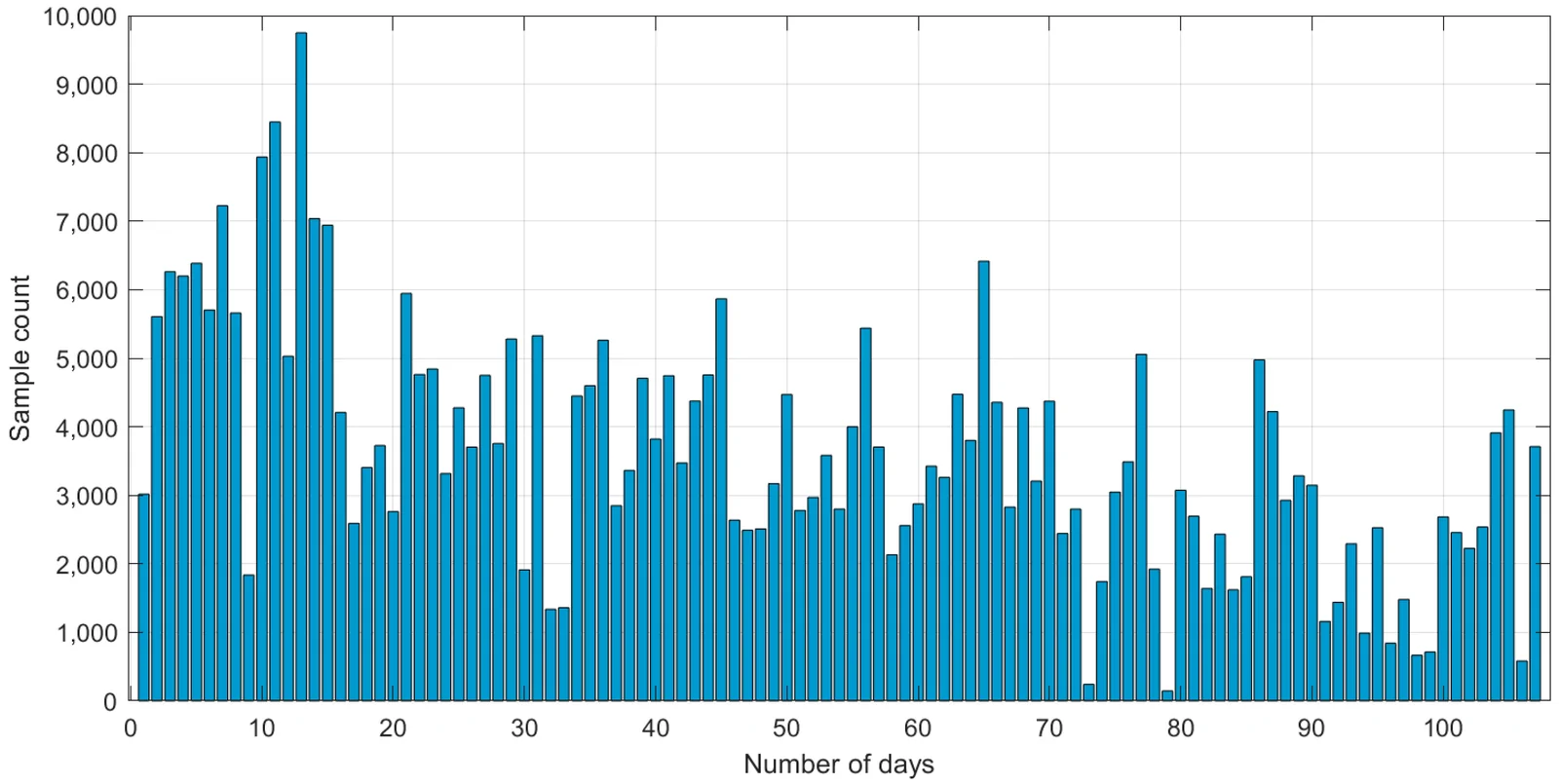
Distribution of sample data.

The dataset includes 388,282 sampling points in total, which were divided into daily subsamples. Because the daily normal operating duration of the unit is different, each subsample contains a different number of sampling points. The final dataset covers 108 days of data, namely 108 subsamples, and the distribution of the sample data is shown in Figure 8.

As can be seen from Figure 7, as the season transitions from autumn to winter, the daily normal operating duration of the unit decreases, and the number of daily sampling points exhibits a corresponding downward trend.

### 4.2. Feature Extraction and Status Assessment

#### 4.2.1. Feature Extraction Results and Analysis

**Figure 9:**
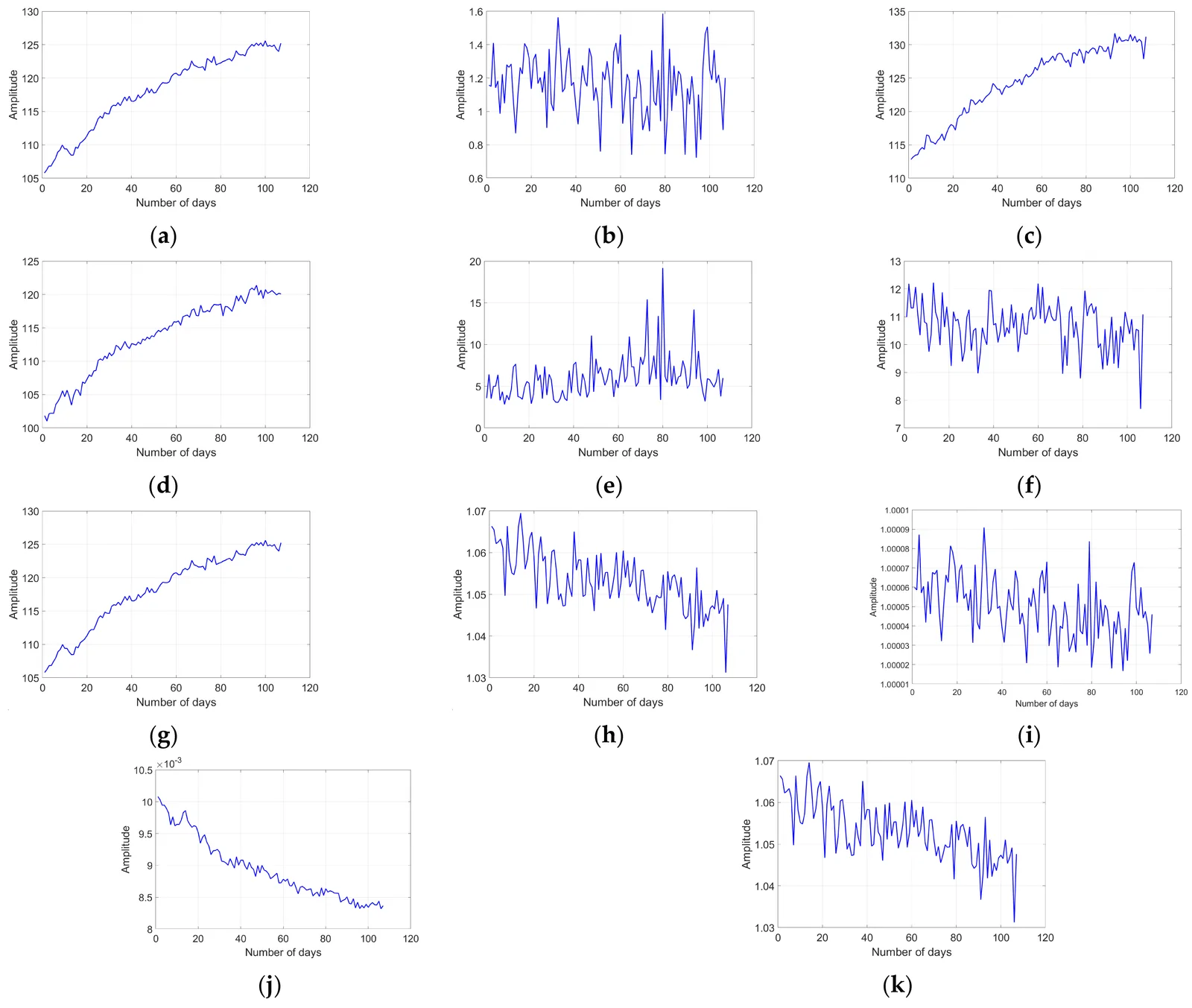
Variation curves of time domain features: (**a**) mean; (**b**) standard deviation; (**c**) maximum; (**d**) minimum; (**e**) kurtosis; (**f**) extreme value; (**g**) root mean square; (**h**) amplitude factor; (**i**) waveform factor; (**j**) margin factor; (**k**) impulse factor.

Time domain features were extracted from the 108 daily subsamples based on Table 1 to construct the assessment samples. The variation curves of each feature are shown in Figure 9.

The mean, maximum, minimum, and root mean square values show a rising pattern during the monitoring period, indicating a general increase in signal energy. The clearance factor and amplitude factor present a downward trend, suggesting that wear may have become more serious. In addition, kurtosis and standard deviation show non-stationary features, which may indicate possible future degradation. The preliminary analysis suggests that the health condition of the unit may decrease toward the end of the year, and further quantitative assessment and evaluation are therefore needed.

#### 4.2.2. Shaft System Status Assessment Results and Analysis

After normalization, the assessment samples were introduced into the entropy weight–coefficient of variation–TOPSIS status assessment model.

**Figure 10:**
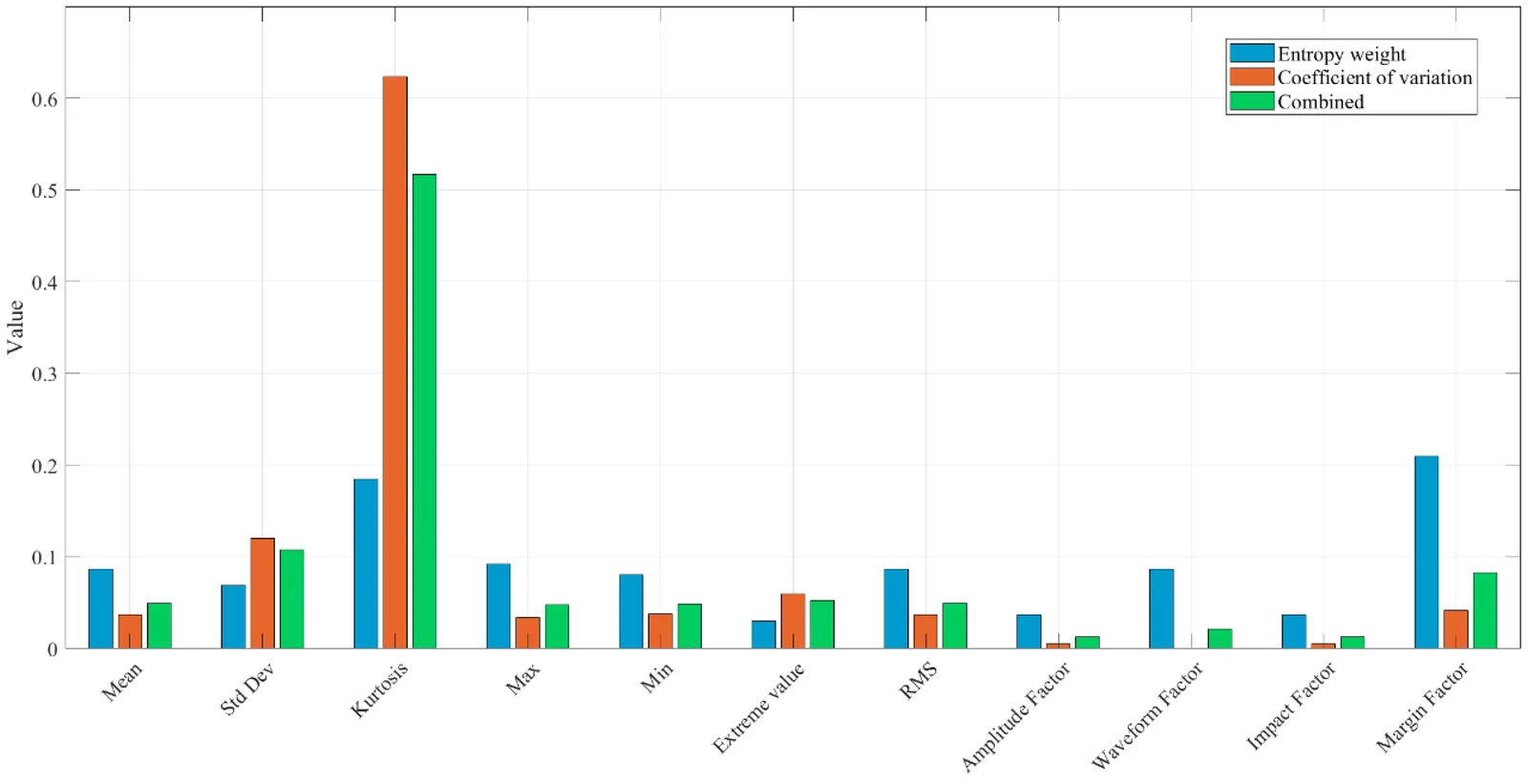
Weights calculated by different methods.

Since the two groups of weights calculated by the entropy weight method and the coefficient of variation method did not pass the Kendall consistency test, the CRITIC method was used to recalculate the weights, and the results are shown in Figure 10.

The relatively high weight of kurtosis shown in Figure 10 reflects its sensitivity to impact-type degradation phenomena such as early rubbing or misalignment. In the shaft system of a hydropower unit, early degradation typically generates intermittent impact forces, causing the vibration signals to deviate from a normal distribution, and kurtosis, with its substantial weight, thus emerges as a key indicator of the shaft system’s health status.

**Figure 11:**
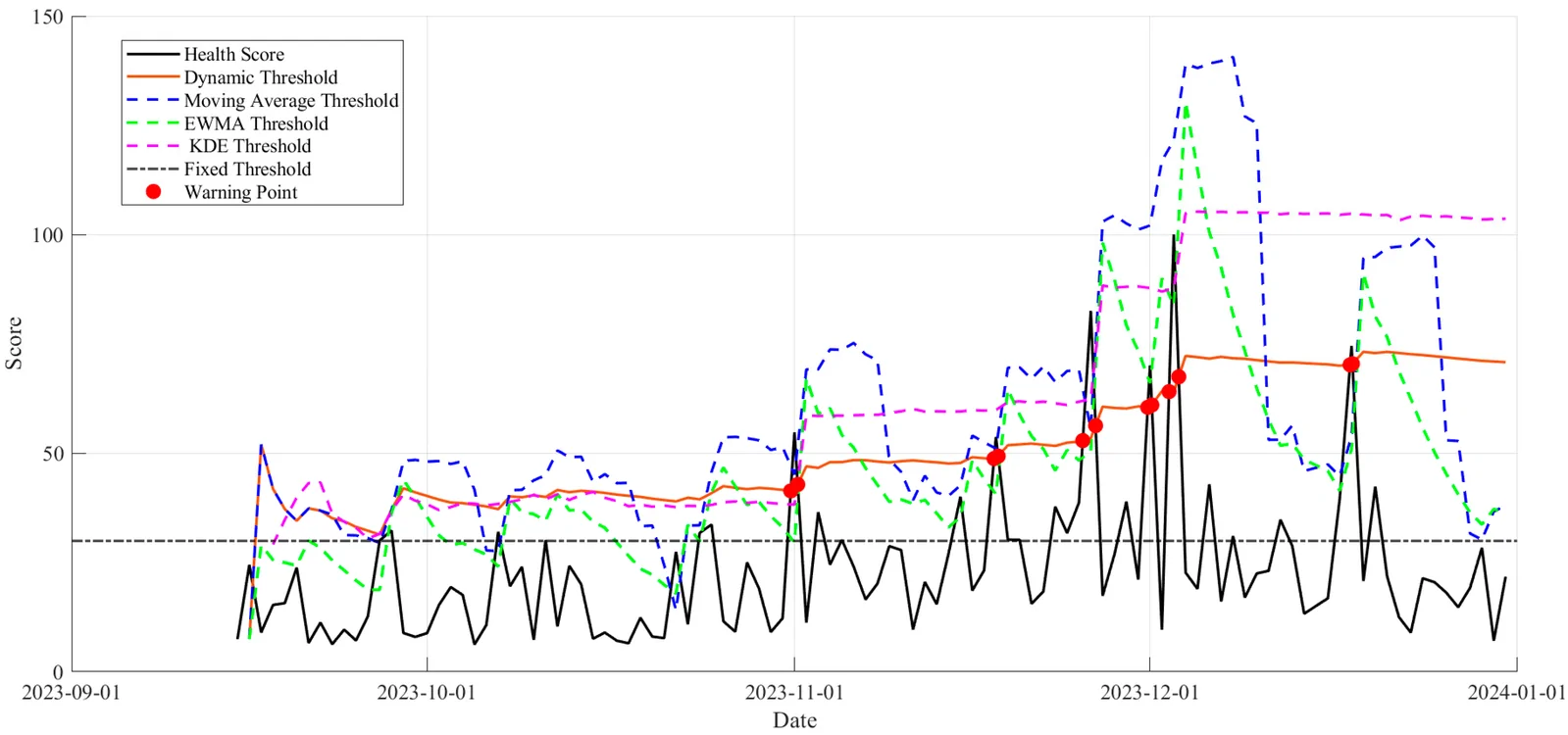
Assessment results of the shaft system operating status.

The shaft system health scores calculated using the entropy weight–coefficient of variation–TOPSIS condition assessment model and the dynamic health threshold method are shown in Figure 11 and Appendix A (Table A1).

As shown in Figure 11, the health score of the unit shows a slowly increasing trend from mid-September to the end of the year. Sporadic warnings are observed in early November, and near the end of November, the health score exceeds the dynamic threshold more often, indicating that the performance of the shaft system has declined compared with the earlier operating stage. This result is consistent with the actual operating condition and should be given attention. A comparison with the actual maintenance records of the unit reveals that a scheduled inspection was carried out in December, during which increased bearing clearance and early rubbing marks on the shaft surface were identified. This confirms that the health assessment correctly identified the onset of degradation at the end of November.

Meanwhile, a comparison was conducted between the dynamic threshold and the moving average, exponentially weighted moving average (EWMA), and kernel density estimation (KDE) methods. The moving average and EWMA methods produced more false alarms in the early stage, whereas the moving average and KDE methods exhibited more missed detections in the later stage. Therefore, the dynamic threshold demonstrated the best stability and reliability. And the dynamic health threshold can be adjusted adaptively according to the data characteristics without manual setting, thus providing higher flexibility than fixed thresholds.

### 4.3. Comparative Experiments on Vibration Swing Signal Prediction Models

#### 4.3.1. Basic Data

**Figure 12:**
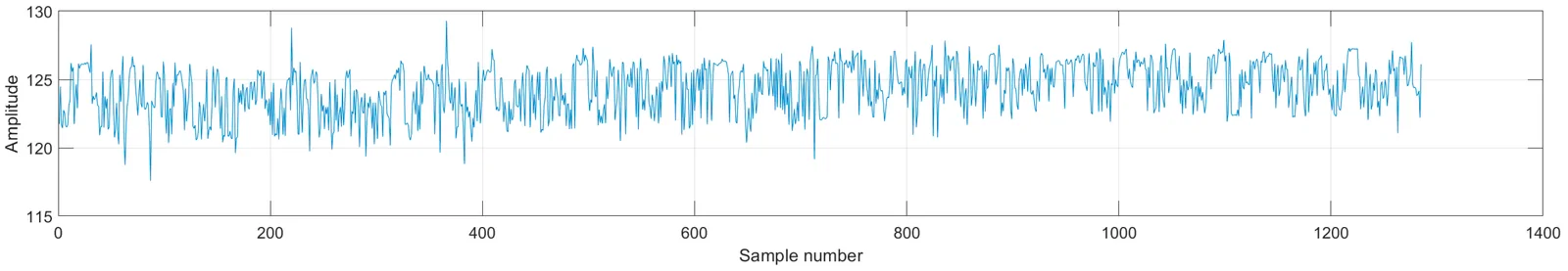
Sampled water head data.

**Figure 13:**
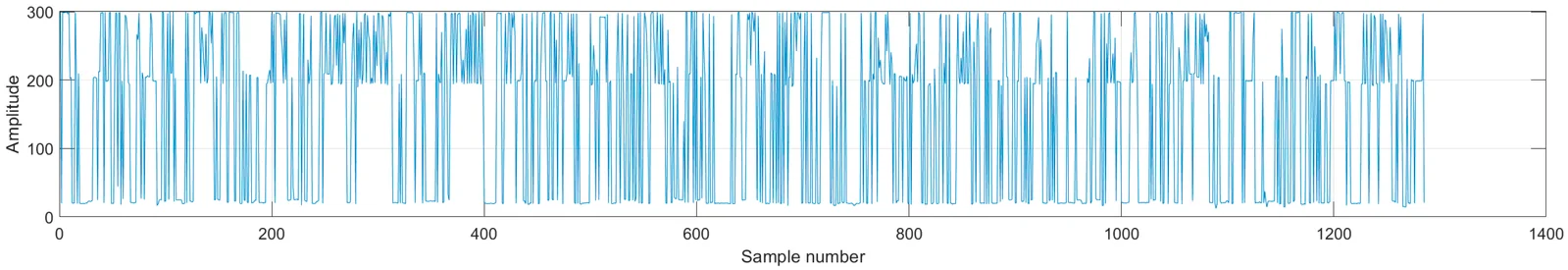
Sampled active power data.

**Figure 14:**
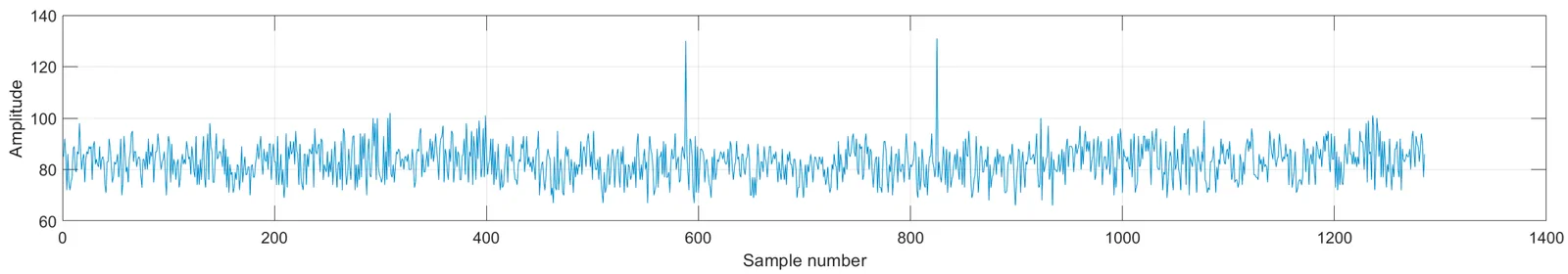
Sampled vibration swing data in the X direction of the lower guide bearing.

The vibration swing signal data in the X direction of the lower guide bearing, recorded at the hydropower station in April, were used for the comparative experiments of prediction models. The data were sampled at 30-min intervals, producing 1286 sampling points in total, and the corresponding water head and active power data were also included. The sampled signals are presented in Figure 12, Figure 13 and Figure 14.

#### 4.3.2. Comparative Experiment of Models Without Signal Decomposition

**Figure 15:**
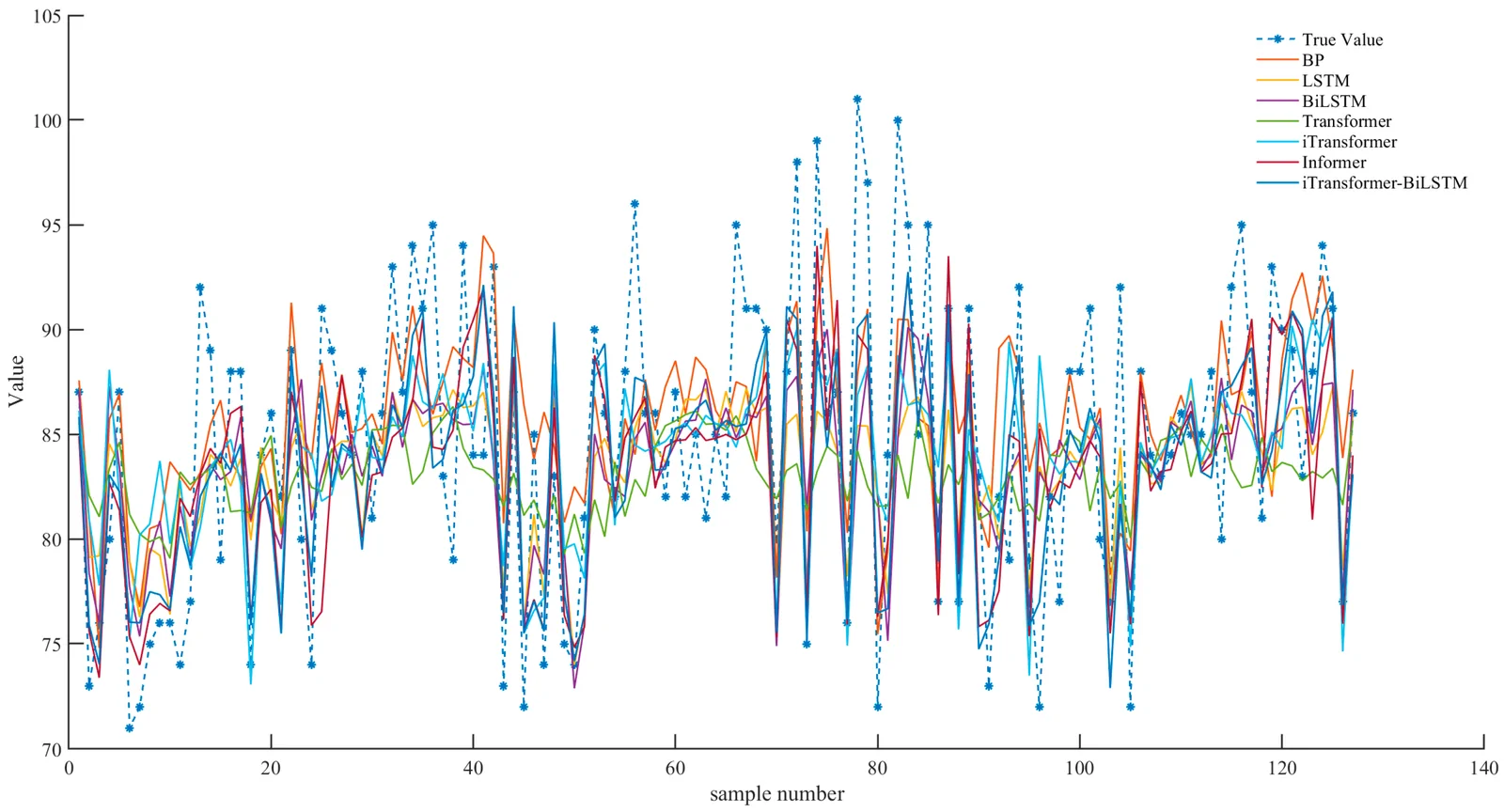
Comparison of predictions from different models without signal decomposition.

Under the condition without signal decomposition, the signal prediction results of each model are presented in Figure 15, and the model performance indicators are listed in Table 2.

The experimental results show that the iTransformer–BiLSTM model performs better than the other comparison models in all indicators, with a coefficient of determination (R^2^) of 0.5402. However, the overall prediction accuracy is still not ideal, which highlights the need for signal decomposition.

#### 4.3.3. Comparative Experiment of Models with Different Mode Decomposition Algorithms

**Figure 16:**
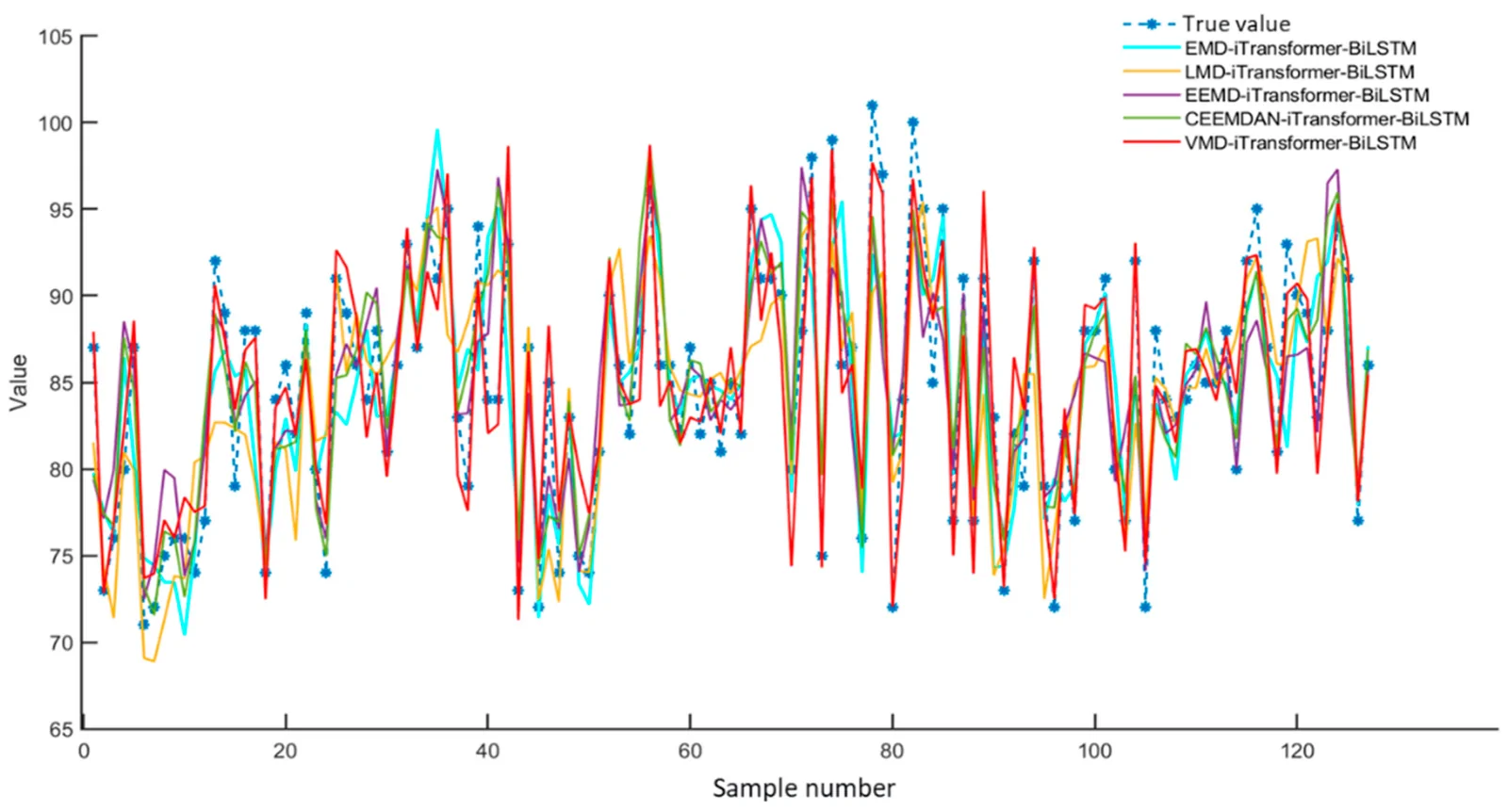
Prediction results based on different mode decomposition algorithms.

VMD was compared with four other signal decomposition methods, namely EMD, LMD, EEMD, and CEEMDAN. All decomposition methods were combined with the iTransformer–BiLSTM prediction model. The prediction results are shown in Figure 16, and the performance indicators of the models are presented in Table 3.

The experimental results indicate that the model using VMD obtains the best prediction effect, reaching an R^2^ of 0.896, which is 65.9% higher than that of the iTransformer–BiLSTM model without signal decomposition.

#### 4.3.4. Comparative Experiment of Models with Different Numbers of Mode Decomposition

**Figure 17:**
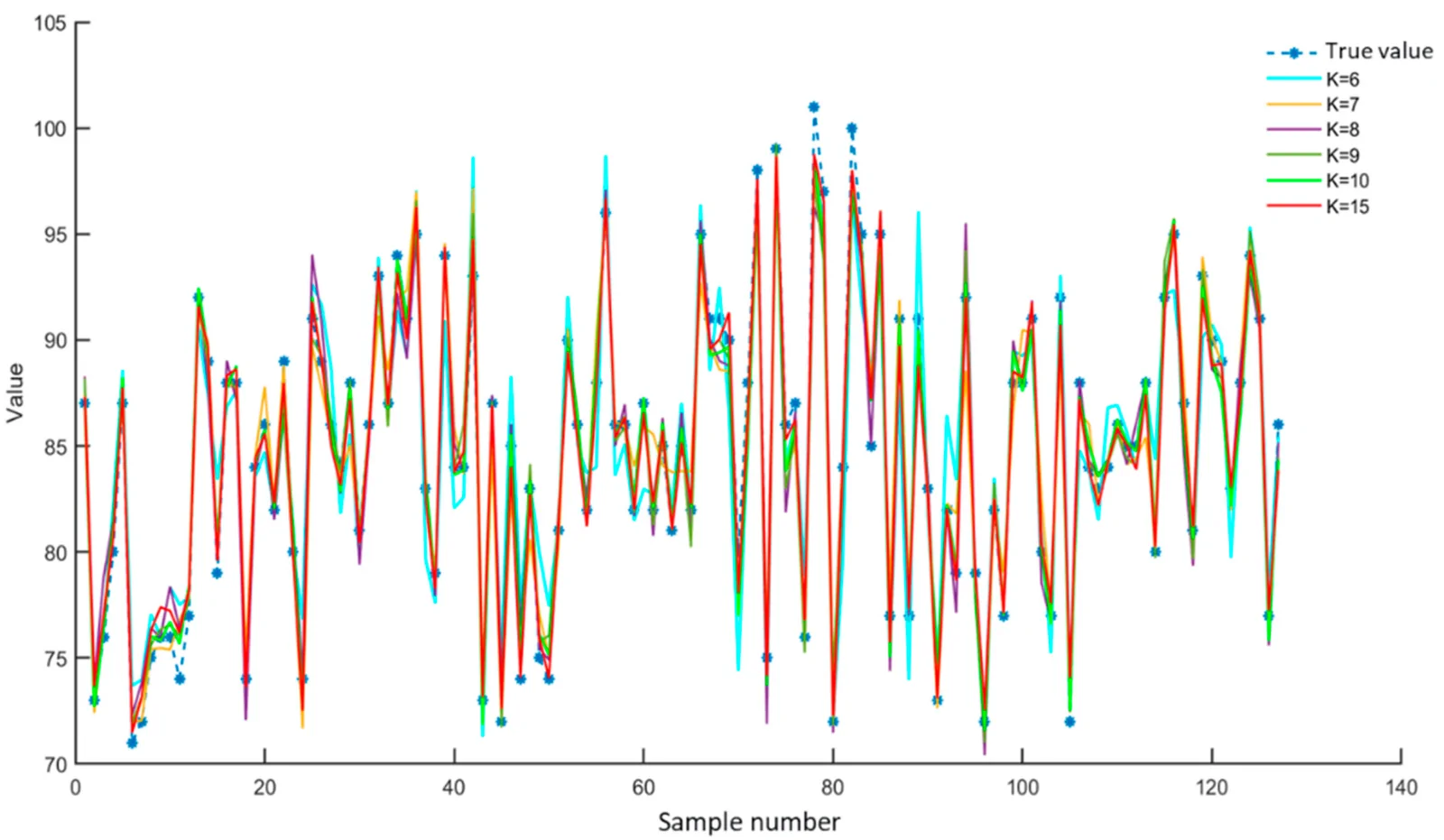
Prediction results based on different mode numbers.

The VMD–iTransformer–BiLSTM model was used to compare the influence of different VMD mode numbers K on the prediction performance of vibration and swing signals. The prediction results are presented in Figure 17, and the model performance indicators are summarized in Table 4.

**Figure 18:**
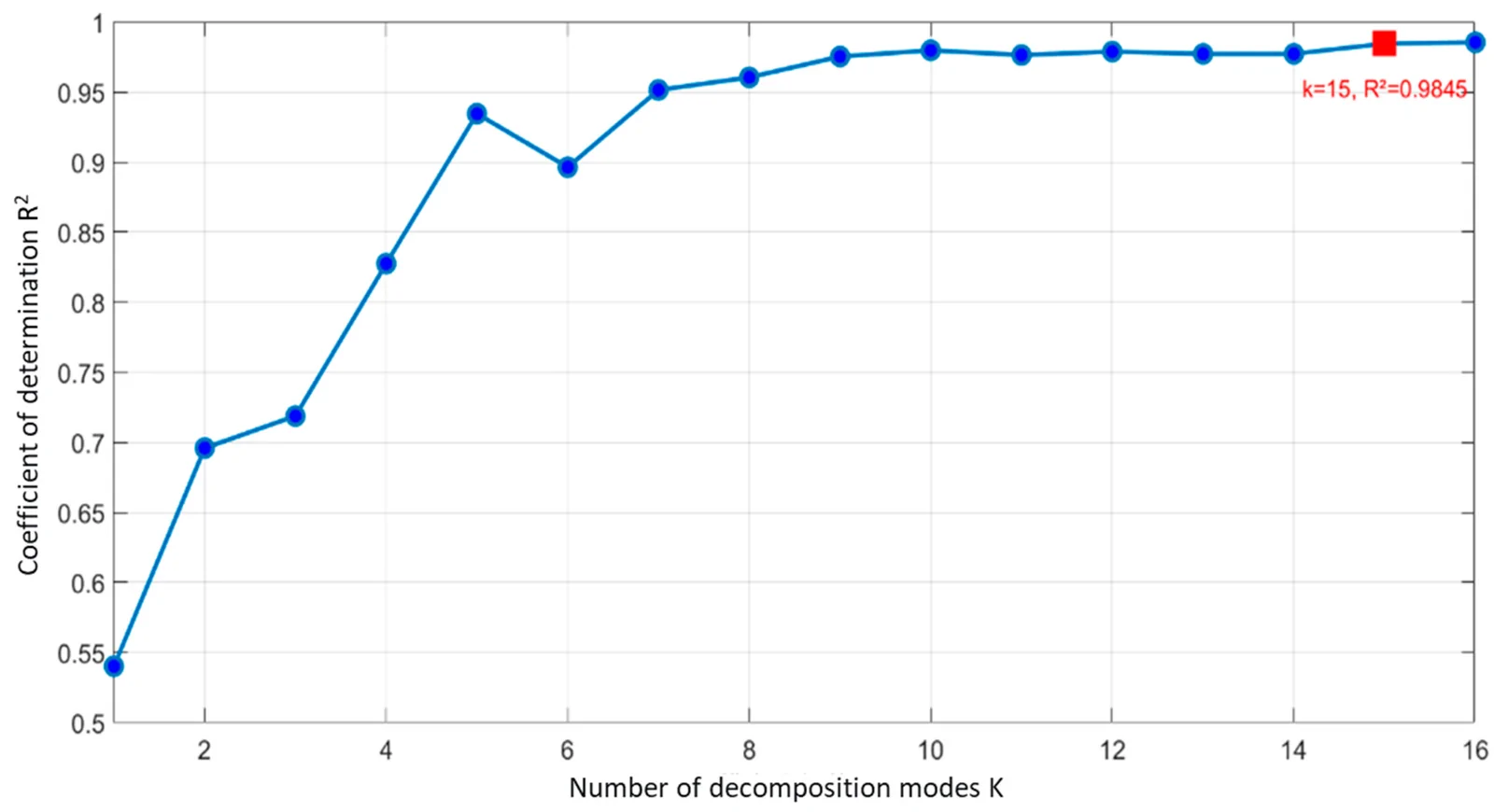
Coefficient of determination versus mode number.

The experimental results demonstrate that prediction performance progressively improves as the mode number K increases. At K = 15, the R^2^ value reaches 0.9845. R^2^ was further computed for K = 1, 2, …, 16, as shown in Figure 18.

As shown in Figure 18, R^2^ presents a general increasing pattern. When K ranges from 9 to 14, R^2^ is maintained within 0.97–0.98. When K = 15, the value is higher than 0.98, whereas at K = 16, the improvement is very small. Therefore, larger K values are not further examined, because an excessively high K may easily lead to mode repetition, overfitting, and longer computational time. By considering both prediction precision and calculation cost, K = 15 is finally chosen.

Through experiments, we ultimately determined the hyperparameters of the VMD–iTransformer–BiLSTM prediction model as follows: the number of mode decomposition K is 15, the number of attention heads is 4, the key dimension is 8, the number of hidden units in the BiLSTM layer is 16, the learning rate is 0.001, and the training time is 119 s.

### 4.4. Vibration Swing Signal Prediction Results and Operating Status Trend Analysis

#### 4.4.1. Vibration Swing Signal Prediction Results and Analysis

**Figure 19:**
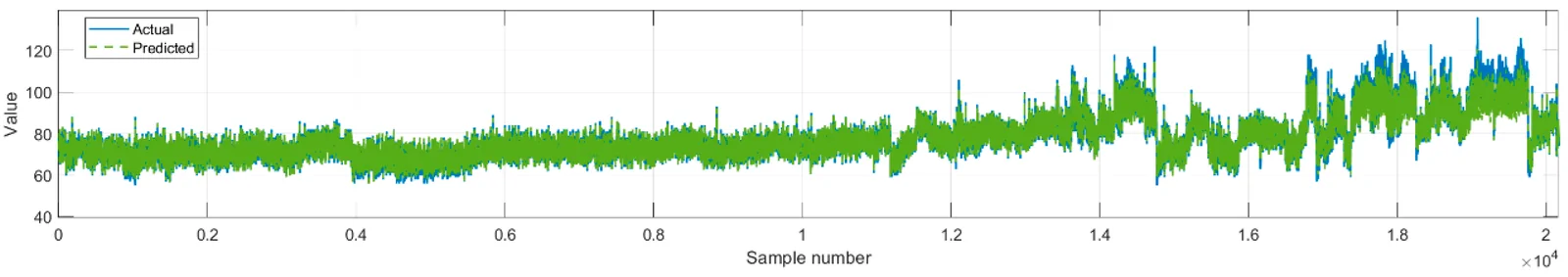
Comparison between actual vibration and swing signals and predicted results.

The vibration and swing signals in the X direction of the lower guide bearing, obtained from the hydropower station during 2023, were adopted for prediction. The VMD–iTransformer–BiLSTM model obtained an R^2^ of 0.9953 on the training set and 0.9774 on the test set, suggesting that overfitting did not occur. The comparison between the measured signals and predicted values from 24 October to 31 December 2023, is shown in Figure 19.

According to the waveform, the predicted values are highly like the actual vibration and swing signals in overall shape, and the differences between the corresponding values at the same time points are limited. The comparison of statistical parameters between the two signals is given in Table 5.

Only minor differences are observed between the predicted and actual vibration and swing signals in terms of statistical parameters, including the mean value (77.87 vs. 77.55), standard deviation (11.08 vs. 9.93), and root mean square (78.66 vs. 78.18), which indicates a good predictive effect.

In this work, the high-accuracy prediction of the raw signal, rather than directly predicting the time domain features, ensures that the recalculated features retain sufficient fidelity for trend analysis. Meanwhile, the feature extraction process itself acts as a noise filter, suppressing the propagation of minor prediction errors.

#### 4.4.2. Operating Status Trend Analysis Based on Predicted Vibration and Swing Signals

**Figure 20:**
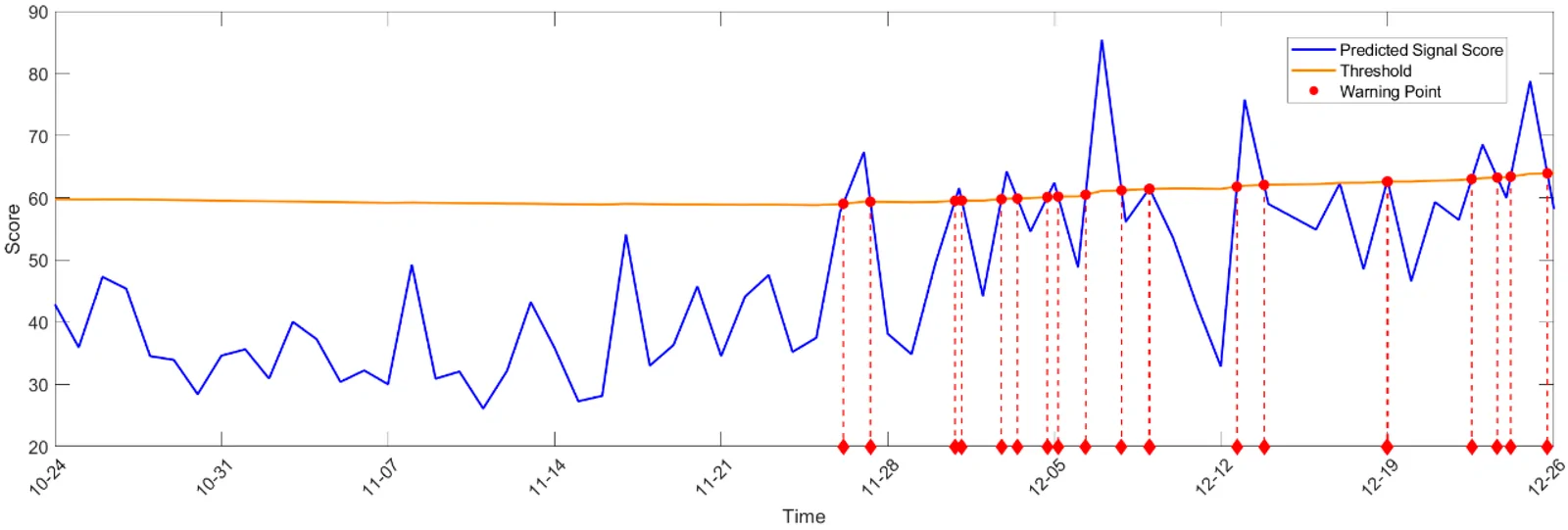
Shaft system health early warning based on predicted vibration and swing signals.

The predicted vibration and swing signals were divided according to date, and the time domain features of each segment were extracted. These features were subsequently introduced into the condition assessment model to calculate the health scores and dynamic health thresholds. The results are presented in Figure 20.

As shown in Figure 20, the first warning point occurs on 27 November 2023, which agrees with the qualitative analysis of the actual vibration and swing signals and with the warning time determined using the health scores calculated from the actual signals. After this date, the health score continuously remains above the dynamic threshold, showing that the shaft system has entered an unhealthy condition and should be given close attention and possible maintenance. This finding confirms the effectiveness of the predicted signals for trend early warning.

## 5. Conclusions and Discussion

### 5.1. Conclusions

To solve the problems of overly simple assessment indicators, poor adaptability of static thresholds, inadequate modeling ability of prediction models for non-stationary signals, and the separation between status assessment and trend prediction, the status assessment and trend prediction of shaft systems in hydroelectric turbine units were studied in this paper by using vibration and swing signals. Based on actual vibration swing data from a Francis turbine hydropower station, a complete technical framework combining status assessment, signal prediction, and trend early warning was constructed, and its effectiveness was verified through a case study.

(1) A shaft system operating status assessment model based on dynamic health score thresholds was proposed. Multidimensional time domain features were extracted from vibration and swing signals to build a measurable indicator system for status assessment. A combined entropy weight–coefficient of variation–TOPSIS model was used to calculate health scores, and dynamic health thresholds were introduced to address the weak adaptability of static thresholds. The case study results show that this method can effectively identify and diagnose the operating status of the shaft system.

(2) A hybrid VMD–iTransformer–BiLSTM model was established for vibration swing signal prediction. The complex signals were decomposed by VMD into several relatively stable IMF components, which reduced the learning difficulty of the model. Global interactions among variables, such as water head and active power, were strengthened by iTransformer through the inverted attention mechanism, while bidirectional temporal dependencies in the sequences were effectively captured by BiLSTM. The case study results indicate that the combined model achieves an R2 of 0.9845, showing much better prediction performance than the comparison models.

(3) A closed-loop procedure from vibration swing signal prediction to shaft system operating status trend analysis was achieved. By calculating health scores and dynamic thresholds from the predicted signals, the case study demonstrates that the warning times are highly consistent with those obtained from the actual signals, confirming the feasibility and effectiveness of the proposed method for predictive maintenance.

### 5.2. Discussion

This paper will discuss the incorporation of additional operational parameters into the health score and the applicability of the method to other units.

(1) The current health score is primarily based on vibration swing signals. Additional operational parameters, such as water level and load fluctuations, could be incorporated in future work. The main challenges include: The increase in feature space dimensionality may require suitable dimensionality reduction techniques to maintain computational efficiency; Introducing new parameters will necessitate recalibrating the evaluation model and the methodology for computing dynamic health thresholds to accommodate changes in the statistical characteristics of the dataset; The expanded feature set may suffer from multicollinearity issues, requiring feature selection and validation to avoid information redundancy. The above extensions will constitute the direction of our future work, aimed at further enhancing the comprehensiveness of the health assessment system.

(2) In this study, only data from a single unit were used. The applicability of the proposed method to other units is discussed below: The proposed method is based on generic time domain vibration features that are readily available in most condition monitoring systems. These features are not specific to any unit, and thus the method possesses the potential for transferability; Transfer learning can be employed to adapt the proposed method to different units. The model can be adjusted by fine-tuning its parameters, and the dynamic thresholds can be adaptively established based on data from the new unit; When units differ substantially in installed capacity, large units may exhibit lower natural frequencies, whereas small units tend to show higher frequency vibration components. In such cases, the sampling frequency should be adjusted accordingly.

Future research will mainly focus on integrating multi-source heterogeneous data, water head prediction, and load prediction to develop a more complete unit health management framework, thereby providing a stronger theoretical basis for the intelligent operation and maintenance of hydroelectric turbine units in new power systems.

## Figures and Tables

**Table 1:** Time domain feature parameters.

No.	Characteristic Parameter	Calculation Method	Physical Meaning and Characterization
1	Mean	$\bar{s}=\frac{1}{N}\sum_{i=1}^{N}s_{i}$	Signal central tendency
2	Standard deviation	$\sigma =\sqrt{\frac{1}{N}\sum_{i=1}^{N}{\left(s_{i}-\bar{s}\right)}^{2}}$	Signal dispersion degree
3	Maximum	$s_{\max}$	Peak level
4	Minimum	$s_{\min}$	Valley level
5	Extreme value	$s_{\max}-s_{\min}$	Fluctuation range
6	Root mean square (RMS)	$RMS=\sqrt{\frac{1}{N}\sum_{i=1}^{N}s_{i}^{2}}$	Effective energy
7	Kurtosis	$\frac{1}{N}\sum_{i=1}^{N}\frac{{\left(s_{i}-\bar{s}\right)}^{4}}{\sigma^{4}}$	Impact characteristics
8	Amplitude factor	$\frac{s_{\max}}{RMS}$	Degree of rubbing
9	Waveform factor	$\frac{RMS}{\frac{1}{N}\sum_{i=1}^{N}\left|s_{i}\right|}$	Waveform shape
10	Impulse factor	$\frac{s_{\max}}{\frac{1}{N}\sum_{i=1}^{N}\left|s_{i}\right|}$	Sudden force
11	Margin factor	$\frac{s_{\max}}{{\left(\frac{1}{N}\sum_{i=1}^{N}\left|s_{i}\right|\right)}^{2}}$	Degree of rubbing

In the table, s denotes a sample point, *N* represents the total number of sample points, *σ* denotes the standard deviation, *RMS* represents the root mean square, $\bar{s}$ is the mean value, $s_{i}$ represents the *i*th sample point, $s_{\max}$ is the maximum value among all sample points, and $s_{\min}$ is the minimum value among the sample points.

**Table 2:** Performance metrics of different models without signal decomposition.

Model	MAE	MAPE	MSE	RMSE	R^2^
BP	4.644	0.0559	33.498	5.788	0.3235
LSTM	4.664	0.0545	32.776	5.725	0.3381
BiLSTM	4.658	0.0545	31.804	5.640	0.3578
Transformer	5.591	0.0654	45.824	6.769	0.0746
iTransformer	4.572	0.0540	32.033	5.660	0.3531
Informer	3.911	0.0455	24.713	4.971	0.5010
iTransformer–BiLSTM	3.854	0.0449	22.770	4.772	0.5402

**Table 3:** Performance metrics of models based on different mode decomposition algorithms.

Model	MAE	MAPE	MSE	RMSE	R^2^
EMD–iTransformer–BiLSTM	3.534	0.0417	20.193	4.494	0.592
LMD–iTransformer–BiLSTM	3.515	0.0416	19.590	4.426	0.604
EEMD–iTransformer–BiLSTM	3.169	0.0373	16.337	4.042	0.670
CEEMDAN–iTransformer–BiLSTM	2.885	0.0341	13.521	3.677	0.727
VMD–iTransformer–BiLSTM	1.841	0.0219	5.133	2.266	0.896

**Table 4:** Performance metrics of models with different mode numbers.

K	MAE	MAPE	MSE	RMSE	R^2^
6	1.841	0.02188	5.133	2.266	0.8964
7	1.230	0.01441	2.405	1.551	0.9514
8	1.078	0.01283	1.959	1.340	0.9605
9	0.821	0.00965	1.221	1.105	0.9753
10	0.762	0.00906	1.005	1.001	0.9797
15	0.688	0.00813	0.766	0.875	0.9845

**Table 5:** Comparison of statistical parameters between actual signals and predicted signal results.

Statistic	Actual Signal	Predicted Result	Difference (Predicted − Actual)
Mean	77.870	77.550	−0.320
Std Dev	11.080	9.930	−1.150
Max	136.000	120.630	−15.370
Min	55.000	55.933	0.933
RMS	78.660	78.181	−0.479
Range	81.000	64.700	−16.300

## Data Availability

The original contributions presented in this study are included in the article. Further inquiries can be directed to the corresponding author.

## Appendix A

**Table A1:** Empirical results of shaft system operational state assessment based on vibration and swing signals of a hydroelectric unit at a certain hydropower station.

Time	Health Score	Dynamic Threshold	Time	Health Score	Dynamic Threshold
15 September 2023	7.4487	7.4487	8 November 2023	20.1535	47.8364
16 September 2023	24.4239	51.9461	9 November 2023	28.7211	48.1186
17 September 2023	8.9920	41.7822	10 November 2023	27.8142	48.3116
18 September 2023	15.2747	37.1612	11 November 2023	9.6605	48.0598
19 September 2023	15.6933	34.5178	12 November 2023	20.5157	47.8729
20 September 2023	23.7935	37.3424	13 November 2023	15.4437	47.5904
21 September 2023	6.5815	36.8351	14 November 2023	26.9970	47.7231
22 September 2023	11.2428	35.0719	15 November 2023	40.0147	49.0394
23 September 2023	6.3038	34.3754	16 November 2023	18.5557	48.7989
24 September 2023	9.6347	33.1063	17 November 2023	23.1769	48.6992
25 September 2023	7.1507	32.2486	18 November 2023	53.6330	51.7693
26 September 2023	12.6631	31.3476	19 November 2023	30.1860	51.9692
27 September 2023	30.0145	37.0650	20 November 2023	30.1471	52.1526
28 September 2023	32.2816	41.9425	21 November 2023	15.5491	51.8765
29 September 2023	8.8692	40.9980	22 November 2023	18.3509	51.6262
30 September 2023	7.9762	40.1805	23 November 2023	37.7257	52.3560
1 October 2023	8.8274	39.3484	24 November 2023	31.6978	52.5990
2 October 2023	15.2551	38.6798	25 November 2023	38.6705	53.3367
3 October 2023	19.3553	38.5450	26 November 2023	82.4970	60.5899
4 October 2023	17.5636	38.1626	27 November 2023	17.3720	60.2867
5 October 2023	6.2455	37.7900	28 November 2023	26.8595	60.1667
6 October 2023	10.6958	37.1656	29 November 2023	38.8935	60.6514
7 October 2023	31.9870	40.0729	30 November 2023	21.0691	60.3929
8 October 2023	19.5543	39.8942	1 December 2023	70.0153	64.1777
9 October 2023	23.9764	40.3285	2 December 2023	9.6284	63.9366
10 October 2023	7.3146	39.9587	3 December 2023	100.0000	72.2045
11 October 2023	30.0876	41.5420	4 December 2023	22.6644	71.8973
12 October 2023	10.3676	41.0384	5 December 2023	18.9549	71.5616
13 October 2023	24.2185	41.3614	6 December 2023	42.8405	71.9801
14 October 2023	19.9888	41.1705	7 December 2023	16.0592	71.6485
15 October 2023	7.5627	40.8695	8 December 2023	31.0120	71.5382
16 October 2023	8.9562	40.4944	9 December 2023	16.9803	71.2168
17 October 2023	7.1131	40.2174	10 December 2023	22.4713	70.9333
18 October 2023	6.5233	39.9697	11 December 2023	23.0677	70.6620
19 October 2023	12.3380	39.5581	12 December 2023	34.7393	70.6873
20 October 2023	8.0496	39.2435	13 December 2023	28.7246	70.5256
21 October 2023	7.6926	38.9475	14 December 2023	13.2743	70.2456
22 October 2023	27.4068	39.7717	16 December 2023	16.8040	69.9537
23 October 2023	10.8598	39.4104	17 December 2023	39.5079	70.1772
24 October 2023	31.7314	40.8290	18 December 2023	74.4671	73.1211
25 October 2023	33.6491	42.4086	19 December 2023	20.7448	72.8348
26 October 2023	11.5352	42.0474	20 December 2023	42.3074	73.1206
27 October 2023	9.1587	41.7379	21 December 2023	21.7950	72.8470
28 October 2023	24.9934	42.0168	22 December 2023	12.4958	72.5892
29 October 2023	19.0500	41.8412	23 December 2023	8.9692	72.3793
30 October 2023	9.0952	41.5641	24 December 2023	21.3848	72.1154
31 October 2023	12.2462	41.2481	25 December 2023	20.4762	71.8500
1 November 2023	54.7143	46.9426	26 December 2023	18.0584	71.5813
2 November 2023	11.2555	46.5930	27 December 2023	14.7558	71.3233
3 November 2023	36.4689	47.9026	28 December 2023	19.1955	71.0643
4 November 2023	24.4826	47.9247	29 December 2023	28.3402	70.9114
5 November 2023	30.1468	48.3817	30 December 2023	7.0981	70.7540
6 November 2023	23.9855	48.3476	31 December 2023	21.7415	70.5186
7 November 2023	16.5192	48.0420			
